# Knowledge, attitude, and practice of Malagasy pharmacy staff regarding the use of topical corticosteroids in children with atopic dermatitis

**DOI:** 10.1016/j.jdin.2024.08.017

**Published:** 2024-09-16

**Authors:** Fandresena Arilala Sendrasoa, Jonah Mamitiana Rakotoniaina, Lala Soavina Ramarozatovo, Fahafahantsoa Rapelanoro Rabenja

**Affiliations:** Department of Dermatology, Faculty of Medicine, University of Antananarivo, Antananarivo, Madagascar

**Keywords:** atopic dermatitis, KAP study, Madagascar, pharmacy staff, topical corticosteroids

*To the Editor:* Atopic dermatitis (AD) is a chronic, pruritic inflammatory skin condition, which usually begins in childhood.^1^ Pharmacists are among the most accessible health care professionals with the potential to greatly affect medication nonadherence through patient education, medication therapy management, and improved access to care.^2^ So, this study aims to evaluate community pharmacists’ knowledge, attitude, and practice (KAP) concerning the use of topical corticosteroids (TC) in AD.

A questionnaire survey was completed by community pharmacists working in 70 pharmacies in Antananarivo and his suburban area, Madagascar (June to December 2022) where 122 officines are available among 211 distributed throughout the island. Community pharmacists, pharmacy technicians, and students in pharmacy who accepted to participate in this study were included. The KAP study made by Kang et al^3^ was used to establish scores of the questionnaire. Each KAP section has questions, each correct response is scored at 1, result is expressed as a percentage.

XLSTAT 2017 was used for the statistical analysis. χ^2^ test was used to analyze qualitative variables, *P* < .05 is considered as statistically significant.

Among 125 pharmacy staff that were sent the questionnaire, 118 (94.4%) replies were received. Respondents’ demographic characteristics and respondents’ knowledge of TC use are shown in Tables I and II, respectively.

**Table I:** Respondents’ demographic characteristics

Demographics	*N* = 118 (%)
Gender	
Male	79 (67)
Female	39 (33)
Age (y)	
<25	6 (94)
25-40	89 (6)
40-55	13
≥55	10
Status of respondents	
Pharmacists	47 (39.8)
Pharmacy technicians	65 (55)
Students in pharmacy	6 (5)
Pharmacists’ years of experience	
<5 y	24 (51)
5-10 y	13 (27.6)
>10 y	10 (21.2)

**Table II:** Knowledge of topical steroids use

Statements	*N* (%)
Drug prescription for AD flares	
Topical corticosteroids	106 (89.8)
Emollient	72 (61)
Antihistamines	104 (88.1)
Oral corticosteroids	64 (54.2)
Administration modalities of topical corticosteroids	
Once daily	109 (92.3)
Twice daily	9 (7.6)
Duration of the TC for AD flares	
<5 d	30 (25.4)
5-10 d	82 (69.4)
>10 d	6 (5)
The quantity of the TC to apply for each application	
Thin layer	113 (95.7)
Thick layer	4 (3.3)
No knowledge	1 (0.8)

KAP score are distributed as follows:

Knowledge: poor (score: <50%); insufficient (score: ≥50 and <65%); moderate (score: ≥65 and <85%); high (score: ≥85%).

Attitude: wrong (score: <50%); insufficient (score: ≥50 and <65%); moderate (score: ≥65 and <85%); right (score: ≥85%).

Practice: poor (score: <50%); inadequate (score: ≥50 and <65%); average (score: ≥65 and <85%); adequate (score: ≥85%).

*AD*, Atopic dermatitis; *TC*, topical corticosteroids.

Regarding pharmacists’ practices, 14.4% stated that they occasionally or often adjusted the prescription by decreasing the duration of TC. For primary counseling in children with AD, 76.3% advise tapering use of TC, and 18% often or systematically informed parents of TC potential side-effects.

No correlation was found between age, gender, years’ experience, and KAP score. Pharmacists had higher level of knowledge (*P* = .01) and attitude score (*P* = .002) compared with pharmacy technicians, but no significative difference in practice score (*P* = .28).

Our results show gaps between perceived and real skills of pharmacy staff in the management of AD. This could be due to a lack of training on AD and/or on TC, as mentioned by Lambrechts et al^4^ that 32.2% of the pharmacists in their study did not remember having lessons about TC, especially the elder ones. It is also possible that standard recommendations for the treatment of AD are not well known. In Japan, in 2016, only 23% of pharmacists have read fully the AD guidelines of the Japan Dermatology Association, and 35% have never read.^5^ In Madagascar, there are no official recommendations adapted to the country’s context regarding the management of AD.

Our results show that 32.2%, 23.5%, and 45.6% of respondents had poor knowledge, wrong attitude, and poor practice concerning TC, respectively. They reflect a lack of training among the pharmacy staff, needing more continued educations in order to improve the management of AD.

## Conflicts of interest

None disclosed.
